# Supplementary material for: Genome-Wide Identification of the AP2/ERF Gene Family and Functional Analysis of PgAP2/ERF187 Under Cold Stress in Panax ginseng C. A. Meyer
Source: Plants (Basel). 2025 Sep 20;14(18):2922. doi: 10.3390/plants14182922 (PMC12473287; doi:10.3390/plants14182922)
Supplement: Supplementary file 1 [file plants-14-02922-s001.zip › Supplementary Figure.pdf]

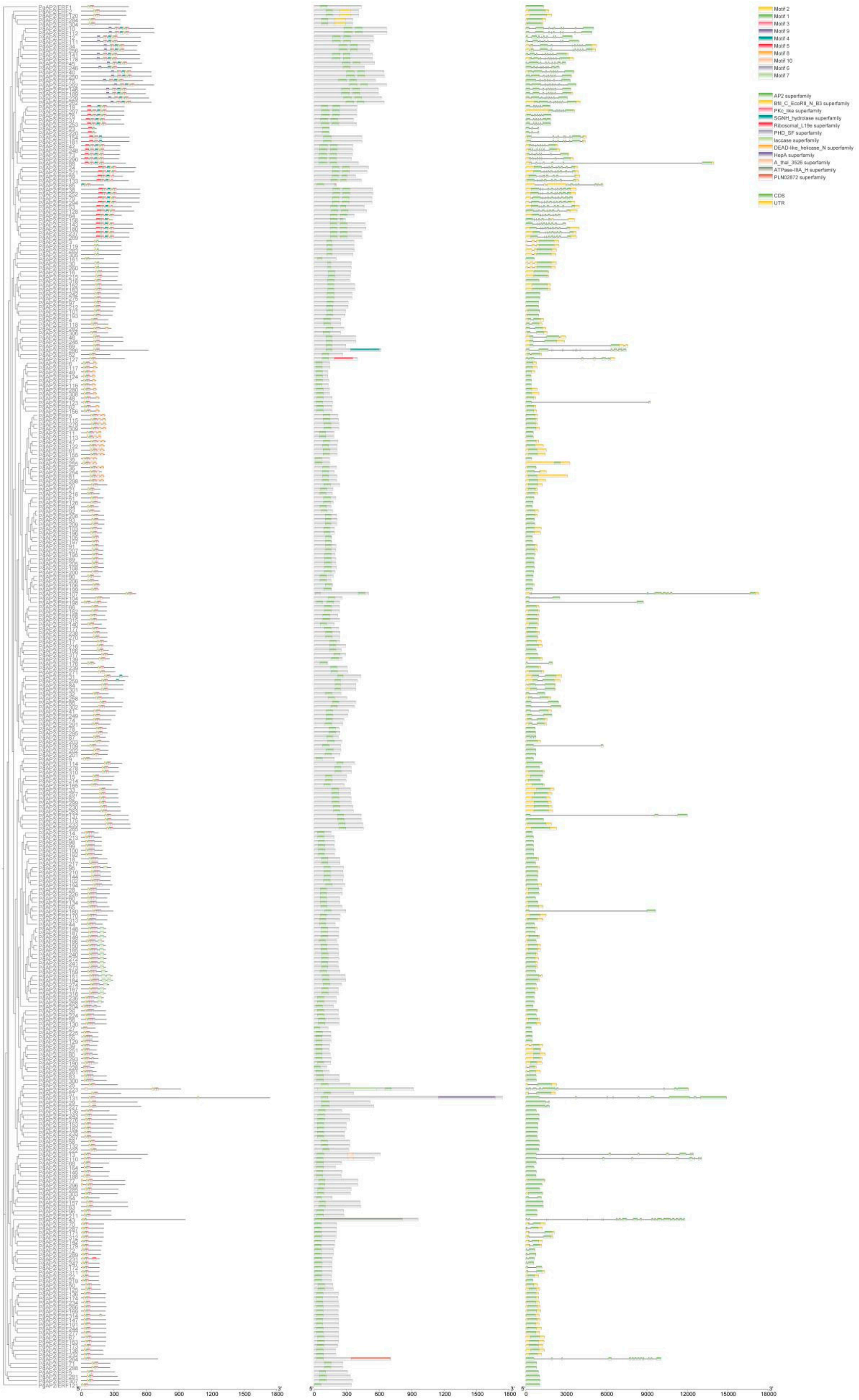

Supplemental Figure S1 Analysis of conserved domains and gene structure of the PgAP2/ERF gene family. (a) Conserved domains. (b) Distribution of AP2 and other domains. (c) Gene structure.

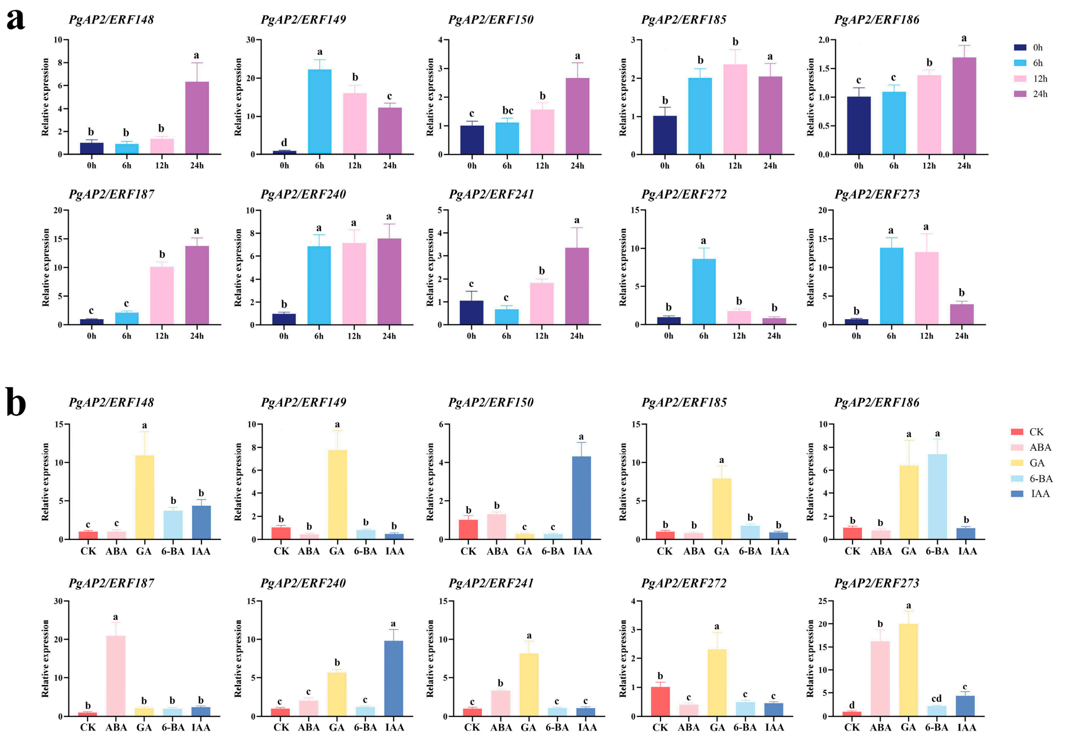

Supplemental Figure S2 qRT-qPCR analysis of PgAP2/ERF expression in P. ginseng under cold stresses and hormone treatments. (a) Expression levels of PgAP2/ERF in cold stress. (b) Expression levels of PgAP2/ERF under different hormone treatments. Note: The values represent mean ± SE (*n* = *3*). Different letters indicate significant differences ($p \leq 0.01$) as determined by one-way ANOVA.

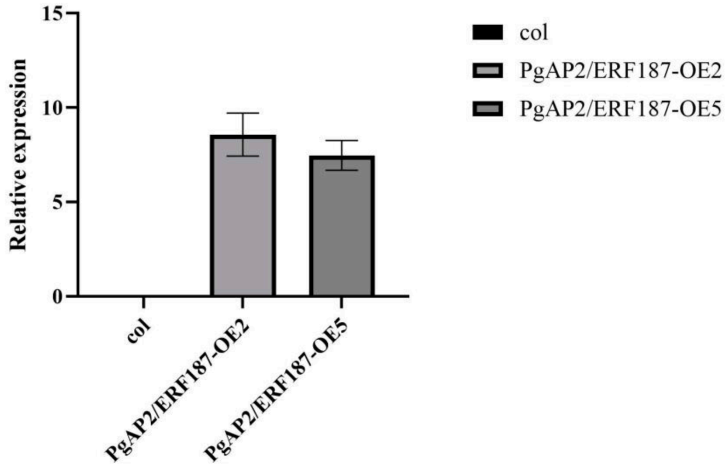

Supplemental Figure S3 Expression levels of *PgAP2/ERF187* in transgenic *A. thaliana* lines.
